# A Systematic Review of Musculoskeletal Mobilization and Manipulation Techniques Used in Veterinary Medicine

**DOI:** 10.3390/ani11102787

**Published:** 2021-09-24

**Authors:** Kevin K. Haussler, Amie L. Hesbach, Laura Romano, Lesley Goff, Anna Bergh

**Affiliations:** 1 Equine Orthopaedic Research Laboratory, Department of Clinical Sciences, College of Veterinary Medicine and Biomedical Sciences, Colorado State University, Fort Collins, CO 80535, USA; 2 EmpowerPhysio, 2585CW The Hague, The Netherlands; amiehesbach@gmail.com; 3 VCA Canada Central Victoria Veterinary Hospital, Victoria, BC V8X 2R3, Canada; laura.romano@vca.com; 4 School of Veterinary Science, Gatton Campus, University of Queensland, Gatton, QLD 4343, Australia; l.goff@uq.edu.au; 5 Department of Clinical Sciences, Swedish University of Agricultural Sciences, 75007 Uppsala, Sweden; Anna.Bergh@slu.se

**Keywords:** manual therapies, mobilization, manipulation, musculoskeletal, osteopathy, chiropractic, dog, horse

## Abstract

**Simple Summary:**

Neck and back pain are common ailments in animals. While there are medical and surgical treatment options available for select patients, conservative care is the most common form of management of pain, stiffness and muscle spasms. Physical therapists, osteopaths and chiropractors use mobilization and manipulation techniques to evaluate and treat muscle and joint problems in both humans and animals, but there seems to be little scientific evidence available to support their use in veterinary medicine. This study reviews the scientific literature with the goal of identifying the clinical indications, dosages, outcome parameters, and efficacy of mobilization and manipulation techniques in dogs and horses. Fourteen articles were included in this review of which 13 were equine and one was a canine study. There was a large variability in the quality of evidence that supports the use of joint mobilization or manipulation in treating pain, stiffness and muscle hypertonicity in horses. Therefore, it was difficult to draw firm conclusions despite all studies reporting positive effects. Future studies need to establish standardized methods to evaluate the optimal dosages of mobilization and manipulation for use in animals.

**Abstract:**

Mobilization and manipulation techniques are often used in small animal and equine practice; however, questions remain concerning indications, dosing and efficacy. A bibliographic search was performed to identify peer-reviewed publications from 1980 to 2020 that evaluated the clinical effects of musculoskeletal mobilization and manipulation techniques in dogs, cats and horses. The search strategy identified 883 papers for review. Inclusion and exclusion criteria were applied. The clinical indications, dosages, outcome parameters, and reported efficacy within each publication were recorded and categorized for comparison with scientific quality assessed according to a standardized grading system. Fourteen articles were included in this systematic review of which 13 were equine and one was a canine study. Seven of these were cohort studies and seven were randomized controlled clinical trials. The canine study involved carpal immobilization-remobilization and all equine studies focused on the effects of passive mobilization (*n* = 5) or manipulation (*n* = 8) of the axial skeleton. Study quality was low (*n* = 4), moderate (*n* = 7), and high (*n* = 3) and included a wide array of outcome parameters with varying levels of efficacy and duration of therapeutic effects, which prevented further meta-analysis. Therefore, it was difficult to draw firm conclusions despite all studies reporting positive effects. Optimal technique indications and dosages need to be determined to improve the standardization of these treatment options.

## 1. Introduction

Manual therapy is defined as the application of the hands to the body with a diagnostic or therapeutic intent [1]. Of the different types of manual techniques that have been used in veterinary medicine, soft tissue massage and joint mobilization or manipulation are the most common techniques applied to animals for the relief or pain, stiffness or muscle hypertonicity [2,3,4,5,6]. Mobilization techniques use graded forces to displace musculoskeletal tissues and can generally be categorized into soft tissue or articular-based approaches [7]. Soft tissue mobilization typically focuses on restoring physiologic motion to the skin and underlying fascia, ligaments, and myotendinous structures with the aim of reducing pain, increasing tissue extensibility, and improving function [8]. Soft tissue mobilization techniques are also used to diagnose and restore normal mobility to neural tissues (i.e., peripheral nerves) [9]. Joint mobilization is characterized as repetitive passive joint movements with the purpose of restoring normal and symmetric articular motion [7]. Manipulation is characterized by the application of a non-repetitive, high-velocity, low-amplitude thrust (HVLA) directed at spinal or appendicular articulations [8].

The incorporation of manual therapies into veterinary practice has become a common approach for addressing neck, back and pelvic pain and dysfunction in both equine and small animal patients [10,11]. Individuals trained in chiropractic, osteopathic and physical therapy techniques use both mobilization and manipulation to address musculoskeletal and neurologic issues in animals [1]. As with most integrative therapies in veterinary medicine, there is often wide-spread clinical use without a strong body of evidence-based support. While there is a growing body of evidence to support the use of mobilization and manipulation techniques in equine practice, there is substantially less published within the small animal literature [12]. General reviews do exist for musculoskeletal mobilization and manipulation use in veterinary medicine; however, no systematic reviews have been completed to date [1,13,14,15]. Analysis of the current scientific literature would provide insights into the clinical indications and effectiveness of mobilization and manipulation in an effort to improve guidelines for their application in managing musculoskeletal disorders. The objective of this systematic review is to describe the literature that has been published relative to mobilization and manipulation techniques in dogs, cats, and horses as a sole treatment modality. The research questions under investigation included: what are the (1) clinical indications, (2) dosages used, (3) outcome parameters, and (4) perceived efficacy of musculoskeletal mobilization and manipulation.

## 2. Materials and Methods

A systematic review process was conducted as outlined in the Preferred Reporting Items for Systematic Reviews and Meta-Analyses (PRISMA) guidelines [16]. Studies were located by professional librarians who performed systematic electronic database searches of the Web of Science, CABI and PubMed in August 2020 for articles published between the years of 1980 and 2020. The following keywords were used in combination with Boolean operators for database searches: dog OR cat OR horse, AND veterinary medicine OR veterinary, AND therap* OR treatment*, AND mobilization OR manipulation OR chiropractic OR osteopathy. Publication date limitations were not implemented. Duplicate references were removed.

Articles were screened for relevance by a single author (KH) and studies unrelated to musculoskeletal mobilization or manipulation were excluded. There were no restrictions regarding the language of publication at the initial search stage. After the first stage of screening, articles deemed potentially relevant were accessed from open-access sources or via inter-library loan via Colorado State University. The resulting list of potential studies was screened by all authors against inclusion and exclusion criteria. The inclusion criteria were publications that must (1) be a primary research publication in a peer-reviewed journal or conference proceedings; (2) report on the treatment effects of a single treatment modality; and (3) describe treatment efficacy for a single clinical disorder or related outcome parameters. The exclusion criteria were studies that were (1) single case reports, textbook chapters, editorials and reviews; (2) involved more than one type of treatment; (3) basic science research exploring mechanisms of action; (4) related to the manual reduction of joint luxations; and (5) focused on rodent models or other animal species (e.g., sheep or pigs). Additional hand searching of the bibliographies of eligible records and review articles were examined for studies not retrieved by database and repository searches.

Each study included in the full-text review was screened for relevance and categorized according to species (canine, feline, or equine) and the type or application of mobilization or manipulation used. Data extracted included the name of first author, year of publication, study design, species, number of animals included, inclusion and exclusion criteria, intervention (dose, interval, duration), controls, follow up period, dropout rate, and treatment results. When more than one outcome parameter was available, the parameter which provided the most clinical relevance was extracted for analysis. The overall study quality was scored using a checklist that included: sample size, confounding factors, selection bias, deviations from planned therapy, dropout rate, blinding, and external validity.

## 3. Results

**Figure 1:**
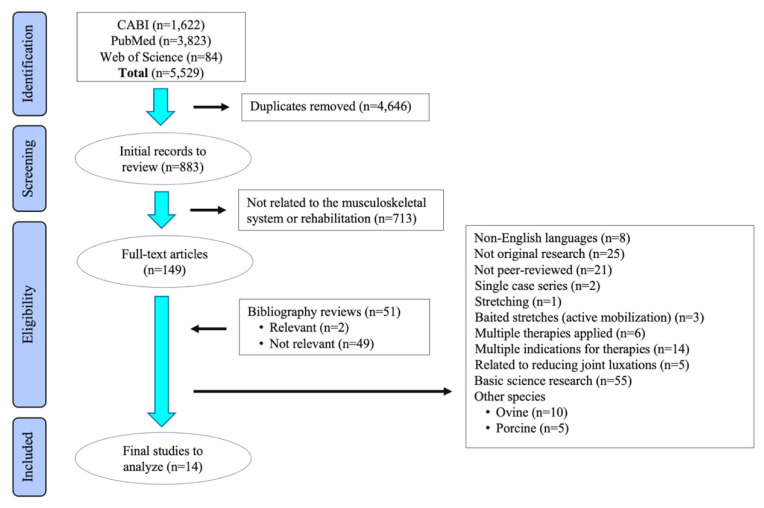
Flow diagram of the stages of the selection process used for identification of studies eligible for systematic review.

A total of 5529 records were identified via the three combined electronic database searches (Figure 1). Following removal of duplicate records, 883 records were screened for relevance to the review. After title and abstract screening, a total of 149 publications that investigated treatment of musculoskeletal issues with mobilization or manipulation techniques were evaluated in full. Hand searching bibliographies from eligible records and review articles provided 51 additional records of which 2 were judged relevant based on inclusion-exclusion criteria. A large proportion of studies were excluded as review articles and textbook chapters; however, the largest number of excluded articles involved basic science research using cats to explore neurophysiologic mechanisms of action for spinal manipulation (*n* = 40) [17,18].

After completion of the selection process, 14 articles were retained of which 13 were equine and 1 was a canine study (Table 1). There were seven experimental studies and seven observational studies. The canine study involved induced carpal immobilization with subsequent remobilization [19]. All equine studies focused on the effects of passive mobilization (*n* = 5) or manipulation (*n* = 8) of the axial skeleton involving either naturally occurring disease (*n* = 12) or induced back pain (*n* = 1). Of these equine studies, two were characterized as whole body or generalized treatments and 11 involved regional or local articular treatments.

Most mobilization studies were cohort-based study designs (5 of 6) with four of these studies being prospective in nature and two retrospective (Table 2). In contrast, the spinal manipulation studies were mostly randomized, controlled clinical trials (6 of 8). Objective outcome parameters were used to assess treatment efficacy in 11 studies and owner surveys were used in the three equine spinal mobilization studies [20,21,22].

The overall quality scores were graded low (*n* = 4), moderate (*n* = 7), and high (*n* = 3) across studies. The three equine osteopathy studies were judged to be of low quality due to their retrospective design and the sole reliance on unspecified owner questionnaires collected 6–18 months after treatment [20,21,22]. The randomized, controlled clinical trials that used objective measures provided the highest quality evidence regarding clinical efficacy [26,27,29].

### 3.1. Clinical Indications

#### 3.1.1. Mobilization

The canine study evaluated the experimental effects of carpal immobilization with remobilization [19]. The equine mobilization studies included three osteopathic reports on the treatment of axial skeleton pain and stiffness [20,21,22]. Medical histories often included behavioral or temperament changes, apprehension when saddled, and reduced ridden performance [22]. Static physical examination findings included the inability to stand squarely on all four limbs, epaxial muscle hypertonicity or atrophy, and signs of pelvic asymmetry. Subjective observation was used to assess changes in stride length, inconsistent limb placement, asymmetric pelvic motion, head and neck elevation combined with trunk lordosis, and the inability to back up in a straight line [22]. A whole body mobilization study in horses with acute back pain assessed the effect of caudal tail traction indicated by active trigger points localized within the longissimus and middle gluteal muscles [24]. In a second whole body mobilization study, the effects of induced caudal weight shifting with manual force applied at the point of the shoulder was evaluated in normal horses [28].

#### 3.1.2. Manipulation

The equine spinal manipulation studies primarily evaluated changes in thoracolumbar nociceptive thresholds (i.e., back pain) and concurrent trunk stiffness and epaxial muscle hypertonicity [10,23,25,26,27,29,30,31]. Experimental studies of equine manipulation included HVLA thrusts applied bilaterally at standardized thoracolumbar locations in actively ridden horses participating in collegiate programs [26,27,29] or with experimentally-induced spinous process pain [30]. In contrast, clinical studies typically addressed HVLA treatment at variable vertebral locations with localized signs of pain or joint stiffness within individual horses [10,23,25,31].

### 3.2. Dosages

#### 3.2.1. Mobilization

In the canine immobilization-remobilization study, carpal flexion, traction, and craniocaudal translation was applied using 3 sets of 20 oscillations, daily for 4 weeks [19]. Equine osteopathic treatments included the use of intravenous sedation (82% of cases) to improve patient compliance and spinal mobilization under general anesthesia (17% of cases) for intransigent pain or dysfunction [21]. The number of treatment sessions using standing sedation averaged 6 treatments (range 1–14) [22]. Cervical mobilization under anesthesia typically involved 1–2 treatment sessions (range 1–6) [20,21]. Post-treatment recommendations often included stall rest and NSAIDs administration for 3–5 days, with restrictions on ridden exercise, and work in hand for 4–8 weeks before return to full work in 4–6 months [21,22]. Caudal tail traction in horses was applied in line with the slope of the croup with three repetitions of a steady applied force of 4.5 kg for 20 s, followed by release for 10 s within a single treatment session [24]. In a second study, caudal weight shifting was induced by applying a caudally-directed manual force to the point of the shoulder in an unweighted forelimb until firm resistance was achieved and the force was then held for 5 s [28].

#### 3.2.2. Manipulation

The frequency for applied HVLA treatments varied from a single session [10,25,27,30,31], to once daily over 3 to 5 days for acute back pain [23], and once weekly for 3 weeks for horses with chronic back pain or stiffness [26,29].

### 3.3. Outcome Parameters

#### 3.3.1. Observation

The clinical examination of horses often included static observation of limb positioning or stance, spinal posture, and epaxial muscle asymmetries [22]. Dynamic observation included gait evaluation in hand while moving in straight lines, circles and while backing up.

#### 3.3.2. Physical Examination

Detailed spinal evaluations of the trunk and pelvis were completed before and after a series of three HVLA treatment sessions in horses with acute back pain [23]. The number of affected thoracolumbar and sacral vertebral segments and the severity of epaxial muscle pain and hypertonicity and segmental trunk stiffness in lateral bending were recorded. Firm digital pressure was used to identify painful sites over thoracolumbar (T4–L6) and sacral (S2–S5) spinous processes and to localize epaxial muscle pain and tone within the thoracolumbar and gluteal regions (T4–S5). Trunk stiffness was identified using low amplitude lateral spinal oscillations applied segmentally at each thoracolumbar (T10–L6) vertebral level. Left-right asymmetries in the prevalence and severity of the spinal examination findings were recorded [23]. In a second spinal manipulation study in horses, myofascial sensitivity localized with a diagnostic acupuncture examination was used [10].

#### 3.3.3. Muscle Tone and Activity

Changes in thoracolumbar epaxial muscle tone were assessed using a tissue indenter in an equine spinal manipulation study [27]. Electromyography (EMG) of the longissimus muscles was recorded during standing and walking to assess changes per and post spinal manipulation [27]. In a second spinal manipulation study, static bioimpedance and dynamic acoustic myography were used to measure changes in epaxial muscle activity in actively ridden horses [10].

#### 3.3.4. Spinal Reflexes

Spinal and pelvic responses to applied digital stimulation were used to assess active spinal mobility, coordination, and core strength in horses with acute back pain that were treated with spinal manipulation [23]. Graded responses to applied truncal stimulation were scored based on the quality, amplitude, and the ability to statically hold the induced postures. Digital stimulation was applied along the ventral midline over the sternum or cranial portion of the linea alba to induce elevation of the cranial thoracic region. Bilateral digital stimulation adjacent the lateral tail head was used to induce a combined reflex of pelvic flexion and trunk elevation (i.e., kyphosis). The response to firm medial compression of both tuber sacrale was scored based on the presence of a pain avoidance response and unilateral or bilateral unlocking of the stifles. Applied axial traction to the tail was used to theoretically assess core stability and neuromuscular coupling of the lumbosacral region [23].

#### 3.3.5. Mechanical Nociceptive Thresholds

The effect of caudal tail traction on back pain in horses was measured using pressure algometry to detect mechanical nociceptive thresholds (MNTs) across lumbopelvic landmarks [24]. Three of the equine spinal manipulation studies also used pressure algometry to measured pre- and post-treatment changes in thoracolumbar MNTs [23,25,26].

#### 3.3.6. Passive Joint Range of Motion

The effects of experimentally induced carpal immobilization-remobilization in dogs was measured with manual goniometry to assess passive joint range of motion and cinematographic analysis of peak flexion and extension angles of the carpus during active walking [19].

#### 3.3.7. Thoracolumbar Flexibility

The vertical displacement of vertebral segments within the thoracolumbar region were measured using a cable extensometer mounted to an overhead rail system during both spinal mobilization and manipulation procedures in horses [29,30]. The inclusion of a pressure-sensitive mat to record applied forces during both spinal mobilization and manipulation allowed the calculation of changes in segmental stiffness pre- and post-treatment.

#### 3.3.8. Motion Analysis

An equine study evaluated the effect of induced caudal weight shifting of the trunk measured cinematographic changes in the vertical displacement and dorsal trunk angles (i.e., lordosis) of skin markers placed along the dorsal midline in normal horses [28]. In a second equine study that evaluated the effects of spinal manipulation, spinal and limb kinematics during overground walking and trotting were measured using high-speed cameras to track three-dimensional skin marker displacements [31].

#### 3.3.9. Visual Analog Scales

Additional clinical measures included visual analog scales (VAS) for assessing overall pain and spinal function in horses with acute back pain [23]. Owners and trainers were asked to score the overall severity of their horse’s back pain based on a VAS that was numbered from 0 to 10, with 0 representing the best case (e.g., no pain) and 10 representing the worst case (e.g., worst possible pain). A VAS was also used for examining the veterinarian’s perception of the global severity of back pain and the overall quality of spinal and pelvic function [23]. 

#### 3.3.10. Owner Surveys of Performance

The equine osteopathic studies relied on owner assessments of improvements in neck mobility and overall performance reported 6–18 months post-treatment [20,21]. The return to regular ridden work as reported by the owner was also used to determine the success of osteopathic treatment [22].

#### 3.3.11. Thermography

Thermographic imaging of the cervical and thoracolumbar regions was used in one of the equine osteopathic studies where temperatures ≥ 1.5 °C cooler than surrounding areas or left-right temperature asymmetries, and loss of the normal dorsal midline thermal demarcation were considered abnormal [22].

#### 3.3.12. Lameness Evaluation

An inertial sensor system was used to assess objective signs of limb lameness on a straight line at a trot in horses treated with spinal manipulation [10].

### 3.4. Clinical Efficacy

#### 3.4.1. Physical Examination

The authors of an equine osteopathic study reported that attempts to classify the degree of pain and dysfunction was considered too subjective to be useful, as the range and severity of clinical signs were too broad and nonspecific [22]. In Western performance horses with acute back pain, a nonsignificant increase (23%) in muscle pain was reported after a series of spinal manipulation sessions applied over 3–5 days, compared to a significant decrease in epaxial muscle pain in horses treated with low-level laser therapy [23]. There was an 83% increase in stiffness as measured by the number of affected vertebral levels in horses treated with a series of HVLA treatment sessions, indicating aggravation of clinical signs. There were no significant changes in the severity of spinous process pain between treatment groups or across treatment sessions [23].

#### 3.4.2. Muscle Tone and Activity

Epaxial muscle tone as measured by a mechanical tissue indenter decreased significantly after a single HVLA treatment session by 13%, compared to 0% change within control horses [27]. Similarly, muscle activity as measured by EMG showed a significant decrease of 21 ± 7% within the treatment group, compared to 6 ± 5% in the control group. A single HVLA treatment session abolished myofascial sensitivity assessed with digital palpation and improved measures of muscle function using static bioimpedance and dynamic acoustic myography for up to 3 days post treatment [10].

#### 3.4.3. Spinal Reflexes

In horses with acute back pain, significant improvements in the quality and amplitudes of spinal motion associated with induced thoracic (28%) and pelvic flexion (28%) reflexes were reported after spinal manipulation [23]. There were no significant treatment effects on the ability to resist axial traction applied to the tail or the response to tuber sacrale compression.

#### 3.4.4. Mechanical Nociceptive Thresholds

Caudal tail traction induced significant changes in MNT values across the 10 lumbopelvic landmarks with an overall increase of 11.6 N/cm^2^ (range 8.7–16.6 N/cm^2^) [24]. Within actively ridden horses without overt signs of back pain, MNT values were significantly increased by 27% after a single instrumented HVLA treatment session 1 week post-treatment, compared to <1% change within both the active and inactive control groups [25]. Spinal manipulation applied weekly for 3 weeks increased MNT values within the treatment group by 11 ± 7% and in the control group by 5 ± 6% [26]. Within the treatment location (i.e., T13–L6), the average increase in MNT values was a 11 ± 4% difference between the treatment versus control horses [26]. In horses with acute back pain, three spinal manipulation sessions produced a significant treatment group effect of 2.3% across pooled MNT values, compared to no significant improvement (−3.9%) when manipulation was combined with low-level laser therapy [23]. However, there was a significant combined manipulation and laser group difference in pooled MNT values from baseline to the third treatment session, but no significant percent change was noted within the spinal manipulation group.

#### 3.4.5. Passive Joint Range of Motion

In dogs, carpal motion after immobilization and remobilization also produced significant increases in passive range of motion amplitudes and peak carpal flexion-extension angles measured during while walking (i.e., active joint range of motion); however, the changes could not be definitively attributed to treatment [19].

#### 3.4.6. Thoracolumbar Flexibility

In horses with induced back pain, vertical trunk displacements increased 15% (range, 7% to 25%) after HVLA, compared to 0% (range, –4% to 7%) in the control group [30]. In actively ridden horses without overt back pain, spinal flexibility as measured by passive vertical displacement increased 16 ± 7% immediately after HVLA treatment across 5 thoracolumbar sites (T14-L6), compared to 0 ± 3% with mobilization alone [29]. However, vertical displacement measured 1 week after spinal manipulation or mobilization, showed an increase in spinal flexibility of 10 ± 5% within the mobilization group, compared to 5 ± 4% in the spinal manipulation group. These findings suggest an immediate effect due to HVLA treatment versus a delayed effect on spinal flexibility associated with spinal mobilization. After 3 weeks of once weekly HVLA treatment sessions, vertical displacement increased by 40% from baseline values versus 19% with spinal mobilization alone [29].

#### 3.4.7. Motion Analysis

Caudal weight shifting in horses caused significant flattening (i.e., reduced lordosis) of the dorsal trunk contour as measured from T10 to L3 with overall changes in vertical displacement of 11 mm (range 1–20 mm) and thoracolumbar angles of 3.4° (range 0.2–7.2°) [28]. In ridden horses with back pain, a single session of spinal manipulation had minor, variable effects on vertebral and pelvic kinematics as measured at the walk and trot [31]. Thoracolumbar and pelvic range of motion tended to increase directly after treatment but was decreased 3 weeks later compared with baseline values. Specific changes included increased thoracolumbar sagittal motion and symmetry of pelvic rotation. No significant changes were noted in stride parameters or cervical vertebral motion patterns [31].

#### 3.4.8. Visual Analog Scales

In horses with acute back pain, veterinarian-derived VAS of back pain severity and spinal function showed no treatment effect over three spinal manipulation sessions, compared to a significant improvement in these parameters within the laser therapy group [23]. Owner reported VAS scores decreased (i.e., reduced back pain) across sessions; however, the changes were not significant for spinal manipulation or combine manipulation and laser therapy.

#### 3.4.9. Owner Surveys of Performance

The treatment of equine neck pain and stiffness using mobilization techniques under anesthesia produced clinical improvement based exclusively on owner reports as early as 2 days, with 95% of horses improved within 2 weeks post treatment [20]. Based on owner reports, 95% of horses are reported to be improved at least 6 months post-treatment using osteopathic techniques with complete resolution in 74% and partial improvement in 26% [21]. In another equine osteopathic study using owner surveys, return to work was reported at 6–12 weeks in 90% of horses, which had all undergone prior unsuccessful conventional treatment [22]. Longer-term follow-up (>12 months) based on rider assessments showed 53% of horses continued in normal work, 31% worked at a lower level, and 16% were unrideable. These authors suggested that the success of treatment depends on owner’s ability to return horses slowly to work, reestablished patterns of normal tissue function with therapeutic exercises or rehabilitation, and repeated treatment using manual therapies [22].

#### 3.4.10. Thermography

In an equine osteopathic study that reported treatment of a wide variety of clinical signs, normal thermographic patterns were noted in horses that had returned to regular work [22].

#### 3.4.11. Lameness Evaluation

There were no significant changes in objective measures of limb lameness in actively ridden horses treated with spinal manipulation [10].

## 4. Discussion

The objective of this systematic review was to analyze the small animal and equine literature for clinical indications, dosages, outcome parameters used, and the perceived efficacy of musculoskeletal mobilization and manipulation techniques. While there is a plethora of review articles addressing the use of manual therapies in veterinary medicine, there is a clear lack of available primary research [7,15]. Joint mobilization and manipulation are known to have effects via biomechanical and neurophysiological mechanisms in humans [32,33]. Numerous basic science studies have evaluated the biomechanical and neurophysiologic mechanisms of spinal manipulation in feline [17,34,35,36], ovine [37,38], and porcine [39,40] models, which made up the largest proportion of excluded literature within this review. Another factor that limited study inclusion was that mobilization and manipulation techniques are often combined with other modalities (e.g., acupuncture) [41,42].

This systematic review reveals that there is a growing body of evidence that supports the use of spinal mobilization and manipulation in horses; however, there remains a critical deficit of published clinical trials in dogs. It is surprising that there are so few studies that have evaluated the effects of joint mobilization or manipulation within the distal limbs of dogs, cats and horses given the high prevalence of appendicular joint disease and associated stiffness that is often addresses with mobilization techniques and the relative ease of which goniometry can be applied in these body regions [43,44,45]. Regrettably, there are also few validated outcome parameters for both dogs and horses for assessing spinal examination findings such as palpable sensitivity, stiffness, and muscle hypertonicity in the clinical setting [23,46]. While functional questionnaires for assessing musculoskeletal or neurologic pain and dysfunction have been validated for use in dogs [47,48,49] and horses [50,51,52,53], these tools have not yet been applied to evaluating the clinical efficacy of mobilization or manipulation techniques.

### 4.1. Quality

The overall study quality scores were low-to-moderate in 11 of 14 studies using the prescribed criteria. Within the observational studies, the largest variability in scoring was within the confounding factors, selection bias and external validity. Within the experimental studies the limiting factor was mostly the lack of blinding.

### 4.2. Treatment Methods

A multimodal rehabilitation approach is common in managing musculoskeletal disorders in veterinary clinical practice and is often judged to have the greatest clinical impact; however, this makes the evaluation of the clinical efficacy of a single treatment modality difficult [41]. Most included studies addressed the effects of either mobilization or manipulation in isolation; however, two equine studies did include both forms of therapies [29,30]. In these two studies, spinal mobilization was required to provide measures of vertical trunk displacement and stiffness across both treatment and control groups whereas, spinal manipulation was only applied with the intent of inducing a treatment effect. Therefore, the treatment group received both spinal mobilization plus HVLA thrusts, which provided insights into the synergistic effects of these combined therapies over 3 sessions at weekly intervals [29]. Both spinal mobilization and manipulation were effective at increasing spinal flexibility; however, HVLA treatment produced larger increases within sessions, whereas the effects of spinal mobilization was delayed as evidenced by changes between sessions. These finding suggested that two possibly different mechanisms of action for spinal mobilization versus manipulation, which is consistent with the human literature [54].

Manual therapy implies using the hand to diagnose or treat. However, instrument-assisted and electromechanical forms of manipulation have also been developed for use in humans to manage musculoskeletal disorders [55]. One of the equine studies included in this review used instrument-assisted manipulation (i.e., Activator), which had a peak effect at the last evaluation 7 days post-treatment [25]. Spinal mobilization and manipulation had peak effects after three treatment sessions at weekly intervals [29]. In humans, comparisons of the effectiveness of mobilization, instrument-assisted and manual manipulation have been reported with no one type of therapy shown to be more effective than the others [56,57]. To date, there have not been any direct comparisons between the efficacy of manual versus instrument-assisted manipulation in animals.

The equine osteopathic studies included in this review incorporated sedation or general anesthesia, which limited any direct comparisons to other spinal mobilization studies that did not use anesthetic agents [20,21,22]. These studies did not clearly report if horses treated under sedation responded differently from horses treated under general anesthesia. General statements were provided by one author that suggested that treating under general anesthesia produced more favorable results and was the preferred technique despite the inherent risks and added costs [21]. However, the authors also reported that general anesthesia was used for horses with more intransigent or chronic issues, which would suggest that the prognosis for these cases might be worse than more subacute cases. Similar indications exist in humans for the use of mobilization or manipulation under anesthesia [58].

### 4.3. Clinical Indications

The subjects included in the studies had naturally occurring axial skeleton pain or stiffness (*n* = 8), no overt back pain (*n* = 4), and experimentally induced pain or stiffness (*n* = 2). In humans, spinal mobilization and manipulation are both reported to have therapeutic effects on neck and back pain [59]. Across the studies included in this review, the treatment areas included the thoracolumbar region (*n* = 11), cervical region (*n* = 3), tail (*n* = 1), and carpus (*n* = 1). The clinical indications across studies included pain (*n* = 8), stiffness (*n* = 6), muscle hypertonicity (*n* = 3) and lameness or poor performance (*n* = 3), which are comparable indications within the human mobilization and manipulation literature [60]. However, it is often difficult to isolate the primary limiting disability or spinal dysfunction specifically to pain, stiffness, or muscle hypertonicity as all three of these clinical issues often occur concurrently to varying degrees within an individual patient [1,7]. The lack of specific musculoskeletal indications prevented the inclusion of several studies in this review [61,62].

### 4.4. Dosages

A single treatment session was reported in 64% (*n* = 9) studies. Recurrent treatment sessions for spinal manipulation included three treatment sessions over 3–5 days [23] or treatment once weekly for three weeks [26,29]. Osteopathic treatment under sedation was applied every 2–6 weeks for an average of 6 treatment sessions [22]. Due to the wide diversity in applied treatment techniques (e.g., trunk displacement, tail traction), treatment sites and the total number of applied mobilizations or HVLA thrusts per treatment session, it is difficult to synthesize the available information into clinically useful dosage recommendations. Some general guidelines have been provided in review articles on joint range of motion and stretching exercises [63]; however, specific guidelines based on this systemic review was not possible due to the paucity of data.

Data from the equine mobilization and manipulation studies suggest that clinical effects are often noted after a single treatment session [10,24,25,27,31]. Most spinal manipulation studies assessed changes in outcome parameters immediately post-treatment; however, short-term effects were reported at 2 to 6 days [10] and up to 1 to 3 weeks after a single HVLA treatment session [25,31]. The effects of a single treatment session are likely to not be useful in formulating practice guidelines where several treatment sessions may be required to achieve the desired therapeutic effects [31].

### 4.5. Outcome Parameters

The outcome parameters reported across studies assessed measures of joint motion (*n* = 6), nociception (*n* = 5), muscle tone or activity (*n* = 3), and performance (*n* = 3). Detailed spinal evaluation procedures have been widely used in veterinary medicine; however, standardization of the techniques and quantitative and qualitative scoring is still in the early stages of development [23,64]. Passive joint range of motion (*n* = 3) and kinematic analysis (*n* = 3) were the most common methods used to evaluate changes in stiffness [19,23,28,29,30,31]. While measures of normal passive joint range of motion has been reported for the appendicular skeleton [44,65], similar measures are not readily available within the axial skeleton due to a diverse array of measurement methods for assessing spinal stiffness [66].

Using pressure algometry to measure MNTs was the most common method used to assess changes in nociception [23,24,25,26]. While there are not well-defined normative MNT values, changes pre-and post-treatment within an individual patient are reported to be reliable [67]. The objective assessment of muscle tone and activity in axial skeleton disorders is challenging [68]. Epaxial muscle tone or activity were assessed using a wide range of methods in this review, which included tonometry and EMG [27], static bioimpedance and dynamic acoustic myography [10], and soft tissue palpation [23].

The ability to return to work based on owner surveys was used in the three equine osteopathic studies [20,21,22]. While owner reports may be useful for global assessments of health or performance, they are limited in quantifying the presence, localization and severity of pain, stiffness or muscle hypertonicity [69,70]. Unfortunately, there are very few validated functional questionnaires or standardized owner surveys in veterinary medicine that have been designed to capture measures of musculoskeletal function and specific responses to applied therapies [47,71]. The appropriate timing and delivery of these tools are also important considerations, which were substandard (i.e., cases over a 19-year period) in most included studies [22].

### 4.6. Perceived Efficacy

Across equine studies, MNT values within the thoracolumbar region increased (i.e., less pain) from 11% to 83%, which suggests a clinically significant improvement [24,25,26]. However, long-term follow up in these three studies was not completed. In one spinal manipulation study, longer-term follow up was provided at 3 weeks post treatment and most of the reported positive clinical effects from 1 h post-treatment had dissipated [31]. Two studies compared spinal manipulation to other forms of therapy [23,25]. In ridden horses without overt signs of back pain, a single treatment of HVLA thrusts was more effective (27%) in reducing pain after 7 days than massage therapy (12%) or 7 days of oral phenylbutazone (8%) [25]. However, in Western performance horses in active competition that presented with signs of acute back pain, epaxial muscle hypertonicity and stiffness, HVLA thrusts produced no significant effects compared to low level laser therapy or combined HVLA and laser treatments [23]. Anecdotally, it appears that spinal manipulation in horses is more effective for treating chronic back pain and stiffness, compared to acute pain syndromes [30]. Similar findings are reported in systematic reviews of spinal mobilization and manipulation for treatment of acute and chronic neck or back pain in humans [72,73].

Most studies reported positive or beneficial effects of musculoskeletal mobilization and manipulation as applied using the described techniques; however, only the experimental studies included control group comparisons. The cohort studies reported changes pre- and post-treatment within individual patients and often did not provide any long-term follow up. In the canine carpal immobilization-remobilization study, there is moderate evidence that repetitive, cyclic joint motion improved passive joint range of motion [19]. Across these three osteopathic studies, the reported efficacy ranged from 75–95% in clinical improvement and return to work in 90% of horses at 6–12 weeks post-treatment, which decreased to 53% after 6 months [22]. However, it is difficult to evaluate true clinical efficacy in the face of low study quality or design.

### 4.7. Limitations

The primary limitation of this systematic review is the large heterogeneity in the indications, applied techniques, treatment protocols, and outcome parameters between studies, which prevented a meaningful interpretation of the overall clinical efficacy of joint mobilization and manipulation. Osteopathic techniques include a diverse array of diagnostic and treatment approaches that range from articular, myofascial, vascular, lymphatic and neural techniques, which makes categorization of the type of applied therapy difficult [74]. It is also difficult and may not be clinically useful to categorize manual therapies into ‘stretching exercises’ versus ‘mobilization’ procedures as the definitions are typically poorly described and there may be a large overlap in the applied techniques [75]. Therefore, it is likely that some studies that might be viewed by others as evaluating ‘mobilization’ were judged by the author to fall more into the ‘stretching’ category and thus were not included in this systematic review.

## 5. Conclusions

There is low-to-moderate quality evidence based on the selected study criteria that various types of joint mobilization or manipulation will reduce pain, stiffness and muscle hypertonicity. The studies are highly heterogeneous in terms of interventions, dosing, duration of treatment, outcome parameters and follow up, which prevented further meta-analysis. Therefore, it is difficult to draw firm conclusions despite all studies reporting positive or therapeutic effects. Future studies need to establish quantitative and qualitative methods to specifically evaluate the effects of mobilization and manipulation, incorporate adequate control groups, provide longer-term follow up, and to include the evaluation of appendicular articulations.

## Figures and Tables

**Table 1:** Summary of musculoskeletal mobilization and manipulation clinical indications and methods.

Indication	Treatment Methods	Species [Citation]
Carpal Stiffness	Joint immobilization-remobilization	Canine [19]
Cervical Pain or stiffness	Osteopathy (with sedation, general anesthesia)	Equine [20,21,22]
Thoracolumbar		
Acute pain	Manipulation	Equine [23]
Chronic pain	Manipulation, Tail traction	Equine [10,24,25,26]
Muscle hypertonicity	Manipulation	Equine [27]
Stiffness	Caudal trunk displacement, Osteopathy (with sedation, general anesthesia), Manipulation	Equine [20,21,22,28,29,30,31]

**Table 2:** Summary of musculoskeletal mobilization and manipulation based on a systematic review of published literature.

Study [Citation]	Study Design	Study Sample	Intervention	Outcome Parameters	Main Results	Study Quality
**Canine Mobilization**						
Olson, 1987 [19]	RCT	**Subjects:** 10 dogs **Inclusion:** Unilateral carpal immobilization with splint × 6 weeks **Exclusion:** NA	**Treatment:** Carpal flexion, traction and craniocaudal translation (*n* = 6): 3 sets of 20 oscillations, once daily × 4 weeks **Control:** No carpal remobilization (*n* = 4)	**Outcomes:** Passive carpal joint ROM, motion analysis of carpal joint angle at walk **Follow up:** 4 weeks Drop out: 2 in control group	Increased carpal passive ROM and flexion-extension joint angles at walk over time (*p* < 0.05), but no group differences **Mobilization:** 140° **Control:** 138°	Moderate
**Equine Mobilization**						
Ahern, 1994 [20]	Cohort, Retrospective	**Subjects:** 86 horses **Inclusion:** Axial skeleton pain **Exclusion:** NA	**Treatment:** Cervical vertebral mobilization under anesthesia—single treatment session **Control:** NA	**Outcomes:** Owner survey **Follow up:** 6–18 months Drop out: 17 of 103 surveys (17%)	Clinical improvement: 95% within 2 weeks; 5% within 6 weeks Maintained improvement: 88% pain free Unsuccessful: 12% return of clinical signs	Low
Pusey, 1995 [22]	Cohort, Retrospective	**Subjects:** 127 horses **Inclusion:** Axial skeleton stiffness **Exclusion:** NA	**Treatment:** Osteopathic treatment under sedation (82%) Mobilization under anesthesia (17%) **Control:** NA Note: Treatment not described	**Outcomes:** Owner survey **Follow up:** >12 months Lost to follow up (2%)	Long-term responses: Improved: 75% No change: 18% Worse: 5%	Low
Colles, 2014 [21]	Cohort	**Subjects:** 51 horses **Inclusion:** Unresponsive chronic lameness or gait abnormality, neck or back stiffness, muscle tone altered, tenderness, thermographic asymmetries (>1.5 °C) **Exclusion:** NA	**Treatment:** Osteopathic treatment under sedation every 2–6 weeks (average 6, range 1–14) Mobilization under anesthesia: Single treatment (67%), 2 treatments (22%), 3 treatments (2%) **Control:** NA	**Outcomes:** Owner survey **Follow up:** 6–12 weeks (short term); 6 months to 7 years (long term) Lost to follow up (37%)	Short-term responses (6–12 weeks): Return to work: 90% Improved performance: 20% Reduced performance: 18% Failed to respond: 10% Long-term responses (>6 months): Return to prior level of work: 53% Reduced level of work: 31% Poor response: 16%	Low
Taylor, 2019 [27,28]	Cohort	**Subjects:** 13 horses **Inclusion:** Normal horses **Exclusion:** Back pain, lameness, analgesics, reduced performance × 6 months	**Treatment:** Caudal truncal displacement—single treatment session **Control:** NA	**Outcomes:** Spinal angles and displacement at 5 thoracolumbar sites **Time points:** Pre- and post-Tx	Increased thoracolumbar flexion (3.4°) Reduced thoracolumbar lordosis (11 mm)	Moderate
Long, 2020 [26]	Cohort	**Subjects:** 11 horses **Inclusion:** Back pain, lameness score 0–2 (out of 5) **Exclusion:** NA	**Treatment:** Caudal tail traction—single treatment session **Control:** NA	**Outcomes:** MNTs at 5 bilateral trunk sites (T18-L3 and pelvis) **Time points:** Pre- and post-Tx	Percent increase in MNT values: Thoracic (83%) Lumbar (50%) Pelvic (52%)	Moderate
**Equine Manipulation**						
Haussler, 2003 [23]	RCT	**Subjects:** 26 actively ridden English collegiate horses **Inclusion:** Back stiffness **Exclusion:** Acute back pain, lameness	**Treatment:** HVLA (*n* = 12)—once weekly × 3 weeks **Control:** No Tx (*n* = 12)	**Outcomes:** MNT at 52 axial skeleton sites **Time points:** Baseline, 7 and 14 days Dropout (*N* = 2, 8%)	Differences in pooled MNT values between treatment and control groups Inside treatment area (T13-L6) = Increased 11 ± 4% (5 of 7 sites) Outside treatment area = Increased 3 ± 8% (2 of 7 sites)	High
Wakeling, 2006 [24,27]	RCT	**Subjects:** 26 collegiate horses **Inclusion:** Epaxial muscle fasciculations, hypertonicity, pain, informed consent **Exclusion:** Overt lameness, concurrent therapies, chronic back problems, history of spinal pathology or foot problems	**Treatment:** HVLA (*n* = 9), Reflex inhibition (*n* = 8)—single treatment sessions **Control:** No Tx (*n* = 9)	**Outcomes:** Epaxial muscle tonometry and EMG: T16 bilaterally **Time points:** Pre- and post-Tx	**HVLA:** Reduced muscle tone (13%), decreased EMG intensity (21%) **Reflex inhibition:** Reduced muscle tone (12%), decreased EMG intensity (18%) **Control:** No change muscle tone (0.3%) or EMG intensity (6%)	High
Haussler, 2007 [28,30]	RCT: Cross-over design	**Subjects:** 10 horses **Inclusion:** Acute back pain model (Steinman pin implantation in spinous processes) **Exclusion:** NA	**Treatment:** HVLA + mobilization (*n* = 10)—single treatment session **Control:** Mobilization only (*n* = 10) Note: Insufficient washout period, concurrent use of NSAIDs	**Outcomes:** Vertical trunk displacements, applied force, stiffness **Time points:** Baseline, post pin implantation, post-Tx, 7-day washout period	**HVLA:** Increased vertical displacement (15%), increased applied force (18%) **Control:** Increased vertical displacement (0%), decreased applied force (2%)	Moderate
Gomez-Alvarez, 2008 [29]	Cohort	**Subjects:** 10 Warmblood horses **Inclusion:** Back pain, asymmetric motion, atrophy **Exclusion:** Lameness, poor prognosis to applied therapy	**Treatment:** HVLA—single treatment session **Control:** NA	**Outcomes:** Vertebral ROM from neck to pelvis; Limb joint angles; Stride length and duration during walk and trot **Time points:** Pre-Tx, 1-h post Tx, 3 weeks post Tx	Stride length: No change Neck angle: No change Limb kinematics: Walk—No change Trot—Increased hip flexion (3°), Increased forelimb flexion (3 cm) FE: Walk—No change; Trot—Increased ROM at T13 and T17 1 -hour post Tx but decreased 3 weeks post Tx LB: Walk—Decreased ROM at T13 and T17 3-weeks post-Tx; Trot—Increased ROM at L3 1-h post-Tx AR: Increased pelvic symmetry	Moderate
Sullivan, 2008 [25,30]	RCT	**Subjects:** 38 horses **Inclusion:** No overt back pain **Exclusion:** Lameness	**Treatment:** Instrumented HVLA (*n* = 8)—single treatment session Massage therapy (*n* = 8)—single treatment session Phenylbutazone (*n* = 7): 2g PO BID × 7 days **Control:** Inactive control (*n* = 7) Active control (*n* = 8)	**Outcomes:** MNTs at 7 thoracolumbar and sacral sites (T3-S2) **Time points:** Baseline, 1, 3 and 7 days post-Tx Note: Owners (21%) refused to allocate horses to HVLA or NSAID groups	Percent increase in MNT values at Day 7: **Instrumented HVLA:** 27% Massage therapy: 12% Phenylbutazone: 8% **Inactive control:** 1% **Active control:** 0%	Moderate
Haussler, 2010 [25,29]	RCT	**Subjects:** 24 actively ridden English collegiate horses **Inclusion:** Normal horses **Exclusion:** Acute back pain, lameness	**Treatment:** HVLA + mobilization (*n* = 12)—once weekly × 3 weeks **Control:** Mobilization only (*n* = 12)	**Outcomes:** Vertical trunk displacements, applied force, stiffness **Time points:** Pre- and post-Tx	Percent change at 3 weeks: **HVLA:** Increased vertical displacement (40%), increased applied force (20%), increased stiffness (7%) **Control:** Increased vertical displacement (19%), decreased applied force (4%), decreased stiffness (15%)	High
Acutt, 2019 [10]	Cohort	**Subjects:** 6 show jumping horses **Inclusion:** Painful response to local palpation **Exclusion:** Lameness, neck or back pain, pathology	**Treatment:** HVLA—single treatment session **Control:** NA	**Outcomes:** Inertial sensor system, static bioimpedance, dynamic acoustic myography, diagnostic acupuncture examination **Time points:** Baseline, 24, 48, and 72 h post-Tx	Percent change over time: Local pain response: Absent immediately post-Tx and at 72 h Lameness: No change Static bioimpedance: Altered at 24 and 72 h post-Tx Dynamic acoustic myography: Altered at walk and trot	Moderate
Haussler, 2020 [31]	RCT	**Subjects:** 61 Western pleasure horses **Inclusion:** Back pain, stiffness, muscle hypertonicity, poor performance **Exclusion:** Lameness >3 (out of 5)	**Treatment:** 3 treatment sessions over 3–5 days HVLA (*n* = 12) HVLA + Low level laser therapy (*n* = 11) Low level laser therapy alone (*n* = 11) Note: Incomplete randomization, concurrent medications or treatments, lacked a negative control	**Outcomes:** Visual analog scale, back pain, epaxial muscle tone, trunk stiffness, MNTs **Time points:** Baseline, 3 sessions over 3–5 days Dropout (44%)	Percent change over time: **HVLA:** Improved thoracic (28%) and pelvic (28%) reflexes, No significant change pain (13%), hypertonicity (17%), stiffness (18%) **HVLA + Laser:** Decreased pain (14%), hypertonicity (55%), stiffness (54%) **Laser alone:** Decreased pain (41%), hypertonicity (20%), stiffness (25%)	Low

RCT = Randomized clinical trial. ROM = Range of motion. Tx = Treatment. MNT = Mechanical nociceptive thresholds. EMG = Electromyography. HVLA = High-velocity, low amplitude. FE = Flexion-extension. LB = Lateral bending. AR = Axial rotation. PO = per os (orally). BID = Twice per day.
